# A case study on SSD to SAD linear acceleartor calibration transition

**DOI:** 10.1002/acm2.70298

**Published:** 2025-10-10

**Authors:** Sharareh Koufigar, Eric Ford, Yulun He, Soren Olsen, Jessica M. Fagerstrom

**Affiliations:** ^1^ Department of Radiation Oncology University of Washington Seattle Washington USA; ^2^ Division of Radiation Oncology Fred Hutch Cancer Center Poulsbo Washington USA

**Keywords:** calibration, linear accelerator, quality assurance

## Abstract

**Purpose:**

Modifying calibration conditions of linear accelerators is infrequent and potentially a high‐risk procedure. This study outlines a systematic approach used to transition a linear accelerator's calibration condition in an active clinical environment from source‐to‐surface (SSD) to source‐to‐axis (SAD), while maintaining treatment accuracy and avoiding interruption of clinical operations.

**Methods:**

A satellite clinic within a university radiation oncology service operated an Elekta Versa HD linear accelerator with SSD calibration, while other system C‐arm accelerators used SAD. With a single installation of the treatment planning system used across all sites, it was decided to convert the machine to SAD calibration. Representative plans with diverse delivery techniques were comprehensively evaluated in advance. Over a single weekend, beams were recommissioned in the treatment planning system (TPS), and output was adjusted per AAPM's TG‐51 protocol. Monitor units (MUs) for on‐treatment patients were scaled manually in the oncology information system, MOSAIQ. Quality assurance (QA) checks, as well as independent peer‐reviewing of each field, were performed to ensure safety and quality for this high‐risk procedure. A retrospective failure modes and effects analysis (FMEA) was subsequently conducted. To evaluate the clinical relevance and broader impact of this work, a targeted survey was conducted via the Wayne State MedPhysUSA LISTSERV.

**Results:**

As a result of the change in output calibration condition, field MU required scaling, ranging from 2.7% to 6.4%. Patient‐specific QA measurements demonstrated consistent gamma pass rates, and both solid‐water phantom and external audit results verified machine output accuracy within 2%. No patient treatments were interrupted during the process. The FMEA identified insufficient expertise and staffing as the highest‐risk failure mode. Survey results indicated that 80% of respondents had never personally performed a calibration transition with patients on treatment, and the majority of respondents characterized the procedure as extremely rare and of higher risk than standard TG‐51 annual QA.

**Conclusions:**

The absolute output calibration condition was successfully transitioned from SSD to SAD without interruptions of patient treatments. Multiple verification steps were implemented to ensure quality and safety. This project contributed to improved standardization across multiple sites of practice.

## 1 INTRODUCTION

A satellite clinic within a university‐affiliated radiation oncology network operates a Versa HD linear accelerator (Elekta AB, Stockholm, Sweden), previously calibrated in source‐to‐surface (SSD) geometry for five photon energies (6 MV, 6 MV FFF, 10 MV, 10 MV FFF, and 18 MV), whereas all other C‐arm linear accelerators in the network use source‐to‐axis (SAD) geometry.

To improve standardization across sites, the Versa HD was converted to SAD geometry for output calibration. This transition was intended to enhance cross‐site consistency, streamline QA procedures, and improve efficiency for physicist cross‐coverage. The project prioritized completion without disrupting patient care, requiting meticulous planning and verification to preserve safety, accuracy, and quality.

Recalibrating a clinical linear accelerator from SSD to SAD is high‐risk due to potential treatment errors, system‐wide dosimetric complexity, and the need for uninterrupted patient care. Currently, no official AAPM Task Group (TG) or Medical Physics Practice Guideline (MPPG) offer formal guidance for such a procedure.

The recalibration was performed by a team of three medical physicists, including the system's chief of physics, and all participated onsite throughout the weekend. The process involved structured planning, controlled implementation, and rigorous verification to ensure clinical and regulatory compliance. This report presents the technical and procedural aspects of transitioning the Versa HD calibration from SSD to SAD, contributing to the broader understanding of calibration transitions in clinical practice.

## 2 METHODS

### 2.1 Planning and risk assessment

The transition of the Versa HD from SSD to SAD calibration geometry was accomplished through a multi‐phase approach. The process was designed to ensure consistency with existing linear accelerators in the university‐affiliated radiation oncology network, while preserving safety and quality and minimizing interruptions to patient care. Planning meetings were held to define the key stages of implementation and to evaluate potential failure modes. These meetings included a comprehensive review of required updates to dosimetric data, treatment planning systems, QA baselines, and patient treatment workflows.

During the planning phase, a sample of patient plans with different delivery and planning techniques was tested to confirm the safety and accuracy of the proposed change. A test machine was created in the treatment planning system, RayStation v.11 (RaySearch Laboratories AB, Stockholm, Sweden) by duplicating the clinical Versa HD beam model and modifying the absolute dose calibration point to reflect the anticipated change. This model was used to recalculate selected patient plans across multiple photon energies and different techniques. Recalculated plans were carefully reviewed to confirm that MU values scaled appropriately relative to the originals. For delivery testing, the recalculated fields were exported to MOSAIQ following the standard workflow, then copied with MUs scaled according to the output change. These fields were subsequently delivered on the machine in QA mode, and measured with an ArcCheck detector array (Sun Nuclear, Melbourne, USA) to verify agreement.

Following completion of preplanning and test measurements, the recalibration process, including all measurements, updates, and validations, was executed over a single weekend to avoid delays in patient treatments. Clear communication among all stakeholders was critical, particularly in ensuring that the dosimetry team was informed not to generate or export treatment plans during the affected period.

### 2.2 Beam measurement and calibration

Tissue maximum ratio (TMR) values were measured and verified in water for all photon beams using the SunSCAN 3D Water scanning system and SNC 125C detectors (Sun Nuclear, Melbourne, USA). The TMR values for the other C‐arm linear accelerators within the university‐affiliated radiation oncology network were tabulated and compared against the measured TMR values for the Versa HD to ensure uniformity across six machines. Output calibration was subsequently performed according to the AAPM Task Group 51 (TG‐51) protocol, using the 3D water tank in conjunction with calibrated farmer‐type ion chamber and electrometer in the SAD setup.^1^, ^2^ Additional verification measurements were acquired using water‐equivalent solid phantom and compared with doses reported by the TPS. Baseline data for daily and monthly QA were re‐measured using the clinic's standard QA equipment, and new baselines were established under the SAD calibration geometry. All results were reviewed to confirm that all parameters remained within acceptable tolerances.

### 2.3 Treatment planning system and dose calculation software recommissioning

All beam model parameters from the previous machine were acceptable and remained unchanged, with the exception of the absolute dose calibration point for the given set up condition. The existing machine model within the treatment planning system was updated with the measured absolute dose calibration. Similarly, the independent dose calculation system in use at the center, RadCalc v.6.4 (Lifeline Software Inc., Tyler, USA), was updated and validated using the revised output reference values to ensure consistency in secondary dose verification.

### 2.4 Patient treatment workflows

Plans for patients currently under treatment or scheduled for upcoming treatment were reviewed to ensure a seamless transition to the new calibration geometry. Because the approach had been tested and verified during the planning phase, plan recalculation in the treatment planning system was not required. Instead, monitor unit (MU) values for each treatment field were adjusted within the MOSAIQ oncology information system (Elekta) based on the post‐recalibration machine output. It was noted that scaling MU for VMAT plans represented a change of required dose rate compared to gantry speed; however, because all photon beams were scaled upward, the monitor units per gantry angle remained above the minimum deliverable threshold, and no delivery limitations related to minimum MU per gantry angle were encountered.^3^

To ensure the accuracy and integrity of the MU scaling, an independent physicist peer‐reviewed each field, including fields with wedges or other modifiers. All fields were reapproved and all changes were documented within MOSAIQ's Navigator Tab environment. Patient‐specific quality assurance measurements were then completed using the ArcCheck, and gamma pass rates were compared to those attained prior to recalibration using the clinical criteria. For both pre‐ and post‐calibration ArcCheck measurements, the passing criteria was implemented of > 90% of diodes above 10% threshold, 2%/2 mm global gamma analysis.^4^, ^5^ MU scaling was selected in place of plan recalculation. While both MU scaling and full plan recalculation carry potential risks, recalculating all affected plans in the TPS would have required substantially more steps across a large number of patients, which could potentially increase the number of opportunities for errors to occur (e.g., incorrect plan selection, unintended plan parameter changes, etc.). In contrast, MU scaling within the oncology information system involved a single, well‐defined calculation step per field, followed by independent peer review of every field and verification through patient‐specific QA. A comprehensive summary of the recalibration process, including dosimetric adjustments and quality assurance outcomes, was compiled and communicated to the larger radiation oncology care delivery team.

### 2.5 Failure modes and effects analysis

A Failure Modes and Effects Analysis (FMEA) was conducted retrospectively to evaluate the recalibration process and identify potential risks that could have been mitigated during planning. Although ideally performed in advance, the post‐implementation FMEA provided valuable insights into areas for improvement in future high‐risk projects, reinforcing the importance of proactive risk assessment in complex clinical workflows. The analysis was carried out by the physicists involved in the recalibration project, including the lead project physicist and the chief physicist of the academic group, with an external physicist observer providing additional oversight. The group collaboratively assigned severity, occurrence, and detectability scores to various potential failure modes based on both the specific preparatory work completed for this project (including tabulating TMR values and TG‐51 factors from similar linear accelerators within the university system for comparison, creating a test machine within the treatment planning system and recalculating patient plans, etc.), and a more general scenario for which this preparatory work was not completed. In addition to the specific conditions of this case study, the more general scenario also assessed scores for a more resource‐limited environment, in which a full team of physicists might not be available based on current national medical physicist staffing patterns.

### 2.6 MedPhysUSA LISTSERV survey

To assess the perceived clinical risk and frequency of SSD‐to‐SAD (or SSD‐to‐SAD) calibration transitions on a linear accelerator with patients on treatment, a short survey was distributed to subscribers of the MedPhysUSA LISTSERV hosted by Wayne State University. The LISTSERV group at the time of survey distribution (April 2025) had approximately 4200 subscribers. The LISTSERV is a forum for medical physics issues with a focus on practices within the United States, though a breakdown of specific demographics within the subscriber group is not available. The survey was administered through Microsoft Forms (Microsoft Corporation, Redmond, WA, USA). It remained open for 30 days and collected responses on clinical experience, perceived risk, and availability of published guidance related to this procedure.

## 3 RESULTS

The recalibration of the Versa HD from SSD to SAD calibration geometry was completed over a weekend with no patient treatments scheduled, with no interruptions to patient treatments. All clinical workflows resumed as scheduled on the following Monday, and no deviations in patient care were reported during the transition period.

### 3.1 Dosimetric adjustments and measurement validation

The recalibration process required scaled adjustments to the output calibration for each photon beam energy. The adjustments required for 6 MV, 6 MV FFF, 10 MV, 10 MV FFF, and 18 MV beams were 2.9%, 2.7%, 3.5%, 3.8%, and 6.4%, respectively. Verification of the recalibration included quality assurance testing using ArcCheck and solid water measurements. A sample of patient plans was re‐delivered on the ArcCheck after recalibration and MU rescaling and compared with the same plans prior to recalibration. Results indicated consistent gamma pass rates across all tested plans, demonstrating that the recalibration had no adverse impact on the delivery accuracy of clinical treatment plans. Pass rates remained above 95% for all analyzed beams, consistent with institutional quality standards. The dose reported in the treatment planning system was verified against the measured dose in solid water for non‐standard field sizes, with acceptable results.

### 3.2 Treatment planning system and secondary dose check validation

Plans recalculated in the recommissioned RayStation treatment planning system and transferred to the recommissioned RadCalc secondary dose calculation software demonstrated improved agreement compared to previous secondary checks. All results were within 2% of expected values. During this transition weekend, 43 patients were on‐treatment on this machine or had plans ready for new starts. Two of these patients were being treated with electrons, and therefore their plans were not impacted. A total of 164 individual fields were rescaled. Each of these fields was crosschecked by a second physicist, with all changes documented within MOSAIQ. For institutions performing a similar transition, it may not be necessary to recalibrate all QA devices prior to performing verification measurements

### 3.3 System‐wide consistency

The recalibrated Versa HD's dosimetric parameters, including adjusted TMR values and reference output, were compared to those of three other SAD‐calibrated linear accelerators within the university system. Ratios were acquired of the absolute dose calibration point for the recalibrated Versa HD to those for the other three linear accelerators. The averages of these ratios for the recalibrated machine were 1.003, 0.995, 1.002, 0.994, And 1.000 for 6 MV, 6 MV FFF, 10 MV, 10 MV FFF, and 18 MV, respectively.

### 3.4 Independent audit

The institution uses the MD Anderson Radiation Dosimetry Services (RSD) thermoluminescent dosimeters (TLDs) as a routine independent audit of output of their machine. RDS was notified of the change in calibration conditions of the machine on file, and a new batch of photon TLDs was requested to be read out after resuming patient treatments. The returned results indicated ratios of absorbed dose determined by RDS to that stated by the institution of 1.02, 1.01, 1.02, 1.01, and 1.01 for 6 MV, 6 MV FFF, 10 MV, 10 MV FFF, and 18 MV, respectively. These ratios were in line with previous results for the institution. Per RDS, agreement within 5% is considered a satisfactory check.

### 3.5 FMEA and risk mitigation

The FMEA results highlighted key areas of risk in the SSD‐to‐SAD calibration transition process, with differences observed between this specific case and a more general scenario without resources for significant preparatory work and without a team of experienced physicists available for second checks. Table 1 summarizes failure modes in both the current case as well as the more general scenario. Risk Priority Numbers (RPN) were calculated using the multiplied severity, occurrence, and detectability scores. The highest RPN failure modes for the general scenario included inadequate expertise and/or staffing and incorrect TMR measurement, primarily due to increased undetectability in a setting with fewer physicists. In contrast, for this specific case, time and resources spent planning as well as redundancy in personnel lowered some of the risks associated with these failure modes. In this case, the highest‐scoring failure mode was judged to be inadequate expertise and staffing.

**TABLE 1:** Summary of FMEA analysis, with failure modes arranged in approximate chronological order.

Category	Task/process	Failure modes	Causes of failure	Effects of Failure	General RPN	Case study RPN
Planning and initial risk assessment	Project planning: Forming a team of physicists	Inadequate expertise or experience, insufficient number of physicists for peer review, etc.	Rushing, lack of staffing, etc.	Inaccurate dose delivered to all patients; patient and staff inconvenience due to delays	210	60
Planning and initial risk assessment	Project planning: machine availability	Process took longer time than scheduled	Unforeseen issues with devices, linac conditions caused by this work (downtime)	Patient and staff inconvenience due to delays	5	5
Beam measurement and calibration	Measure TMR using water tank	Incorrect TMR measured	Incorrect measurement conditions, including issues with tank, scanning control software, ion chamber, etc.	Inaccurate dose calculations for all future planned patients	80	16
Beam measurement and calibration	TG‐51 and independent verification (e.g., OSLD)	Inaccurate dose calibration	Incorrect measurement conditions, including issues with tank, scanning control software, ion chamber, etc., incorrect process followed	Inaccurate dose delivered to all patients	40	8
Beam measurement and calibration	Reestablishment of daily/monthly QA baseline reference values	Wrong reference values set	Missing energies to rebaseline, incorrect calibration conditions	Patient inconvenience due to morning QA failing and requiring physics intervention	16	16
Treatment planning system and dose calculation software recommissioning	Update and validate machine model in TPS	Wrong calibration value entered	Rushing, inexperience with TPS software commissioning interface, mistyping	Inaccurate dose calculations for all future planned patients	200	40
Treatment planning system and dose calculation software recommissioning	Update calibration factors for independent secondary dose calculation software	Forgetting to update, updated factors not input correctly	Rushing, inexperience with secondary check software commissioning interface, mistyping, incorrect validation setup	MUs mismatch; staff inconvenience	80	16
Patient treatment workflows	Scaling MUs in MOSAIQ R&V system for patients currently on‐treatment	Forgetting to scale, scaling by the wrong factor, no peer review, unable to scale MUs	Mathematical error (multiplication vs. division), mistyping, minimum MU/segment limitation violated	Inaccurate dose delivered to patient; staff/patient inconvenience for beam stoppages	360	72
Patient treatment workflows	Verify/update patient‐specific QA methods, if needed	Forgetting to verify/update	Rushing, incorrect calibration conditions, wrong test plans to verify calibration	Inaccurate QA results, staff inconvenience due to unnecessary replans	16	16
Project finalization	Step‐by‐step documentation of the recalibration project	Not done thoroughly/correctly	Rushing, lack of time, inattention	Staff inconvenience for future projects when needing to confirm sections of the current project or replicate the procedure	48	16

### 3.6 MedPhysUSA LISTSERV survey

Data was collected from 69 survey respondents. Survey responses indicated respondents considered SSD‐to‐SAD calibration transitions during active treatment as a high‐risk and low‐occurrence clinical task. Most respondents indicated limited experience with such transitions and perceived a lack of formal guidance in the literature. Additional survey results, including response distributions and representative free‐text response comments, are provided as Supplementary Information.

## 4 DISCUSSION

The transition of a linear accelerator from SSD to SAD calibration geometry represents a high‐risk undertaking due to the potential for errors that could directly impact patient safety and treatment efficacy. As such, the application of a risk‐based framework, such as the AAPM Task Group 100 (TG‐100) methodology,^6^ is strongly recommended for similar projects. The TG‐100 approach emphasizes proactive risk assessment through FMEA, structured quality management, and the prioritization of mitigation strategies to address identified risks. By systematically analyzing each step of the recalibration process, potential failure modes—such as inaccuracies in dosimetric measurements, treatment planning system updates, or monitor unit scaling—can be identified and addressed before implementation.

Overall, the FMEA completed for this project suggests that meticulous work pre‐planning and allocation of sufficient institutional resources reduced the risk of major calibration errors. However, in a setting with limited staffing and/or inadequate preparation, significant risks emerged that could lead to incorrect dose delivery. These findings reinforce the need for structured risk mitigation strategies, including standardized verification steps, redundancy in personnel for critical tasks, and thorough peer review of calibration outputs.

The results of this study demonstrate that transitioning a linear accelerator from SSD to SAD calibration can be achieved efficiently while maintaining high standards of dosimetric accuracy and patient safety. The successful recalibration of the Versa HD without treatment interruptions underscores the importance of a well‐structured approach that includes preemptive planning, comprehensive quality assurance, and interdisciplinary collaboration. The consistency in gamma pass rates and agreement with independent dosimetric audits confirm that the transition did not introduce clinically significant discrepancies in dose delivery. Furthermore, the alignment of recalibrated dosimetric parameters with those of other SAD‐calibrated machines within the system enhances the robustness of cross‐site operations, reducing variability in physicist workflows and improving standardization across the larger institutional network.

Beyond the technical and procedural aspects of the recalibration, this study highlights broader implications for clinical practice. The comparison of risk priority numbers between the well‐resourced environment of this study and a more resource‐limited scenario highlights the critical role of institutional support, adequate staffing, and rigorous peer review in maintaining patient safety during high‐risk procedures. Institutions with limited personnel or experience in calibration transitions may benefit from additional safeguards, such as external peer review or phased implementation strategies to reduce the likelihood of errors.

The MedPhysUSA LISTSERV survey indicated that such transitions during active treatment are rarely performed and are widely perceived by survey respondents as higher risk than routine TG‐51 QA. Most respondents reported limited personal experience and noted a lack of formal guidance in published literature or professional documentation. These findings align with the results of the FMEA, which identified inadequate expertise and staffing as key risk drivers. In combination, the survey responses and risk analysis emphasize the need for structured planning and clear recommendations to support safe implementation of calibration transitions in clinical settings. This case highlights the interaction between procedural design, institutional resources, and clinical safety in high‐risk machine calibration transitions. The desired outcomes achieved (maintenance of treatment accuracy, uninterrupted patient care, and consistent QA results) were supported by multiple layers of redundancy, proactive planning, and a large, experienced physics team. In a resource‐limited environment, the absence of these safeguards could magnify the potential for undetected errors, as reflected by the higher RPN values in the generalized FMEA scenario.

## 5 CONCLUSIONS

The successful recalibration of a linear accelerator from SSD to SAD calibration geometry demonstrates the feasibility of standardizing dosimetric workflows across a multi‐site radiation oncology system. This project achieved its objectives of aligning the Versa HD with institutional calibration practices while ensuring continuity in patient care and treatment accuracy. The recalibration process, completed within a compressed weekend timeline, maintained treatment quality without disruptions. Dosimetric adjustments for all photon beam energies were implemented and validated against institutional standards, achieving agreement within 2% across recalibrated systems. Quality assurance testing, including ArcCheck gamma analysis, confirmed consistent pass rates exceeding 95% before and after recalibration, underscoring the reliability of the adjusted setup.

Recommissioning of the treatment planning system and secondary dose calculation software enhanced system‐wide consistency, with recalculated plans showing improved agreement compared to prior secondary checks. This standardization facilitated improved cross‐site clinical operations. Additionally, comparisons of the recalibrated Versa HD's dosimetric parameters with other SAD‐calibrated linear accelerators in the system verified uniformity across clinical sites. An independent audit confirmed the results.

This study highlights the importance of careful planning, interdisciplinary collaboration, and rigorous quality assurance in calibration transitions. By aligning calibration geometries across all clinical sites, the institution achieved enhanced operational efficiency and streamlined workflows while maintaining high standards of patient safety.

## AUTHOR CONTRIBUTION

All authors contributed to the creation and writing of this manuscript.

## CONFLICTS OF INTEREST STATEMENT

The authors report no relevant conflicts of interest.

## Supporting information



Supporting Information

## Data Availability

The data that supports the findings of this study are available in the supporting information of this article.
