# Supplementary material for: A case study on SSD to SAD linear acceleartor calibration transition
Source: J Appl Clin Med Phys. 2025 Oct 10;26(10):e70298. doi: 10.1002/acm2.70298 (PMC12513801; doi:10.1002/acm2.70298)
Supplement: Supplementary file 1 — Supporting Information [file ACM2-26-e70298-s001.pdf]

Linear accelerator calibration geometry survey results

We conducted a survey targeting subscribers of the Wayne State MedPhysUSA LISTSERV from April to May, 2025. Over a 30-day period, we received 69 responses via Microsoft Forms. Key findings from the survey include:

- 80% of respondents reported they had never personally performed an SSD-to-SAD or SAD-to-SSD transition on a linear accelerator in clinical service while patients were on treatment, 19% reported they had, and 1% were unsure if they had personally performed such a transition.
- When asked to quantify how common they perceived this transition is in clinical practice, 78% classified such transitions as 'extremely rare,' 14% classified such transitions as ‘somewhat rare,’ and 1% classified such transition as ‘somewhat common.’ No respondents classified such transitions as ‘extremely common or routine,’ and 6% of respondents said they were not sure.
- When asked to compare the risk level of such a transition to standard annual TG-51 QA, 29% classified this transition as ‘very high risk,’ 49% classified this transition as ‘high risk,’ 16% classified this transition as ‘medium risk,’ and 6% classified this transition as ‘low risk.’
- 83% reported never having seen published guidance on SSD/SAD calibration transitions, 16% reported that they were not sure if they had seen published guidance previously, and 1% reported that they had seen published guidance.

Free-text responses emphasized caution, suggested MPPG guidance, and highlighted the absence of clear documentation for such transitions. Selected comments include:

- *“I have a mixed clinic with one old Trilogy set up for SAD calibration that I would like to convert to SSD calibration, but I'm reluctant due to the perceived risk of doing something wrong! Are there AAPM guidelines on how to handle this? Thanks for your investigation—I would like to see your results.”*
- *“I am interested in the results of this survey as I am considering making a change.”*
- *“Excellent question—I would love to see a procedure to follow for such a process.”*
- *“I suggest you propose an MPPG on this topic. There is a medium to high risk when changing the calibration geometry.”*
- *“I would only do this transition on an accelerator that does not have ongoing patient treatments—during major overhauls, etc.”*
- *“I would like to convert to SSD calibration but am reluctant due to perceived risk I might do something wrong! Are there AAPM guidelines on how to handle this?”*
- *“The transition of patients under treatment was much harder and more time consuming than the actual change of output on the linac.”*
- *“With patients on treatment it's extremely high stakes. I'm sure it can be done, I just wouldn't feel comfortable doing it at my own clinic.”*
- *“There would need to be a very compelling reason to do this. What would be the gain that is worth the risks to the patients of something going wrong? Secondarily, what is the gain that is worth all of the effort from physics and dosimetry to do this?”*
- *“Inevitably something is going to be affected that you aren't aware of or hadn't thought about before. I haven't gone through the transition personally but that always seems to be the case. Go through a dry run of all the things, patient plan transition, monthly QA, annual QA, etc. With patients on treatment its extremely high stakes. I'm sure it can be done, I just wouldn't feel comfortable doing it at my own clinic. Good luck!”*
- *“I would not take this type of change lightly, and give your team lots of time to prepare. The transition of patients under treatment was much harder and more time consuming than the actual change of output on the linac.”*
